# Supplementary material for: Global hypo-methylation in a proportion of glioblastoma enriched for an astrocytic signature is associated with increased invasion and altered immune landscape
Source: eLife. 2022 Nov 22;11:e77335. doi: 10.7554/eLife.77335 (PMC9681209; doi:10.7554/eLife.77335)
Supplement: Figure 2—source data 1. [file elife-77335-fig2-data1.zip › Figure_2_source_data_1/Figure_2I_J/homerResults/motif14.similar.html]

motif14

## Information for motif14

T
C
A
G
C
T
A
G
T
C
A
G
T
C
G
A
A
T
C
G
T
G
A
C
G
A
T
C
G
C
A
T
A
T
C
G
T
A
C
G
A
T
G
C
C
G
T
A
T
A
C
G
C
A
T
G
T
C
A
G
C
G
T
A
A
C
T
G
A
T
G
C
C
G
T
A
A
T
C
G
T
C
G
A
C
T
G
A
C
T
A
G
A
C
T
G
T
A
G
C
G
T
C
A
A
G
T
C
A
T
C
G
T
A
C
G
T
G
C
A
  
Reverse Opposite:  

A
C
G
T
A
T
G
C
T
A
G
C
T
C
A
G
C
A
G
T
A
T
C
G
T
G
A
C
G
A
T
C
G
A
C
T
A
G
C
T
T
A
G
C
G
A
C
T
T
A
C
G
T
G
A
C
G
C
A
T
A
G
T
C
G
T
A
C
A
T
G
C
C
G
A
T
T
A
C
G
A
T
G
C
T
A
G
C
C
G
T
A
C
T
A
G
A
C
T
G
T
A
G
C
A
G
C
T
A
G
T
C
G
A
T
C
A
G
T
C
  

|  |  |
| --- | --- |
| p-value: | 1e-40 |
| log p-value: | -9.242e+01 |
| Information Content per bp: | 1.492 |
| Number of Target Sequences with motif | 58.0 |
| Percentage of Target Sequences with motif | 7.99% |
| Number of Background Sequences with motif | 2.7 |
| Percentage of Background Sequences with motif | 0.97% |
| Average Position of motif in Targets | 99.0 +/- 49.4bp |
| Average Position of motif in Background | 117.0 +/- 38.5bp |
| Strand Bias (log2 ratio + to - strand density) | -0.1 |
| Multiplicity (# of sites on avg that occur together) | 1.19 |
| Motif File: | file (matrix) reverse opposite |

### Similar de novo motifs found

|  |  |  |  |  |  |  |  |
| --- | --- | --- | --- | --- | --- | --- | --- |
| Rank | Match Score | Redundant Motif | P-value | log P-value | % of Targets | % of Background | Motif file |
| 1 | 0.649 | A C T G A C T G T A C G T G C A A T G C T G A C A T G C A C T G A T C G T A C G A T G C G T A C A T C G C A T G A T C G C T A G A T C G T A G C T G A C A T C G T C A G T C A G T A C G A T G C T A G C T G C A A T C G A T C G T A C G C T A G T A G C A G C T A T C G A G C T A T G C G A C T A G T C A T G C A T C G A T C G | 1e-28 | -64.941444 | 4.96% | 0.66% | motif file (matrix) |
| 2 | 0.817 | C A T G A C T G T C A G T C G A A T G C T A G C A T G C A G C T A T G C T A C G A T G C G C A T A T C G A C T G T C A G T C G A A C T G A G T C G T C A T A C G T G C A C T G A T A C G A G T C T A G C | 1e-27 | -62.255640 | 4.82% | 0.69% | motif file (matrix) |
| 3 | 0.604 | A T C G C A G T T A C G C T G A C A T G T C G A A T G C T A G C G C A T A T C G T A G C C G A T A T C G T C A G T A C G C T G A T A C G A T G C G T C A T A C G C T G A C A T G T C A G T A G C T A G C T G A C G C A T T C A G T A C G T C G A T A C G A T G C A T G C G C T A A T G C | 1e-21 | -50.619210 | 5.51% | 1.03% | motif file (matrix) |
| 4 | 0.638 | T A C G T A C G C T A G A T C G T G A C A T G C A G C T T A G C T A C G T A G C G T C A A T C G T C A G T C A G C G T A A T C G A T G C G T C A A T C G T C G A C T G A T A C G G A C T T A G C G T C A A T G C A T G C T A C G T C G A A T G C G A C T T A C G G A C T A T G C G C A T G A T C A G T C C A T G T A C G T A C G T C G A T G A C G A T C T A G C C A G T | 1e-19 | -44.716513 | 7.02% | 1.46% | motif file (matrix) |
| 5 | 0.732 | A C T G A T C G T A C G T C G A A T G C G A T C A T G C G A C T A G T C A T C G G A T C G C T A A T G C C A T G T C A G C G T A A T C G A G T C C T G A A C T G T G C A C G T A A T C G C A G T A T G C T G C A T A G C A T G C C A T G G T C A A T G C A G C T A T C G C G A T A T G C | 1e-15 | -34.862620 | 3.31% | 0.43% | motif file (matrix) |
